# Herpes Zoster in Hematological Disorders: Pathogenesis, Risk Stratification, and Emerging Strategies for Prevention and Immunization

**DOI:** 10.1111/ejh.70049

**Published:** 2025-10-07

**Authors:** Enrica Antonia Martino, Ernesto Vigna, Antonella Bruzzese, Nicola Amodio, Eugenio Lucia, Virginia Olivito, Caterina Labanca, Santino Caserta, Francesco Mendicino, Fortunato Morabito, Massimo Gentile

**Affiliations:** ^1^ Haematology Unit, Department of Onco‐Haematology AO of Cosenza Cosenza Italy; ^2^ Department of Experimental and Clinical Medicine University of Catanzaro Catanzaro Italy; ^3^ AIL Sezione di Cosenza Cosenza Italy; ^4^ Department of Pharmacy, Health and Nutritional Science University of Calabria Rende Italy

**Keywords:** antiviral prophylaxis, hematological malignancies, herpes zoster, immunocompromised hosts, mRNA vaccines, recombinant zoster vaccine, stem cell transplant, varicella‐zoster virus

## Abstract

**Background:**

Herpes zoster (HZ), resulting from reactivation of latent varicella‐zoster virus (VZV), imposes a significant burden on immunocompromised patients, particularly those with hematological malignancies and recipients of hematopoietic stem cell transplants (HSCT). These populations face markedly increased risks of severe complications, including disseminated disease and postherpetic neuralgia.

**Objective:**

This review examines the pathogenesis, epidemiology, risk factors, and evolving preventive strategies for HZ in patients with hematological disorders, with an emphasis on antiviral prophylaxis and vaccination.

**Methods:**

Relevant data from recent clinical trials, observational studies, and international guidelines were critically reviewed to evaluate the burden of HZ and the effectiveness of prophylactic interventions in immunocompromised populations.

**Results:**

Immunosuppression—whether due to the underlying disease or post‐transplant immune reconstitution—compromises VZV‐specific cellular immunity, thereby increasing HZ susceptibility. Incidence rates in high‐risk groups, such as HSCT recipients or patients treated with proteasome inhibitors, JAK2 inhibitors, or CAR‐T therapies, may exceed 30–60 per 1000 person‐years. Antiviral prophylaxis remains a fundamental preventive approach. The adjuvanted recombinant zoster vaccine (aRZV) demonstrates 68%–87% efficacy even in heavily immunosuppressed individuals. Emerging vaccine platforms, including mRNA‐based formulations, offer promising improvements in immunogenicity and scalability.

**Conclusion:**

Patients with hematological conditions are particularly vulnerable to HZ. Effective prevention requires a tailored combination of antiviral prophylaxis and aRZV immunization. The development of mRNA‐based vaccines may further enhance preventive strategies for this at‐risk population.

## 1 Introduction

Herpes zoster (HZ) results from the reactivation of latent varicella‐zoster virus (VZV), which establishes lifelong latency in the dorsal root or cranial nerve ganglia following primary infection, typically acquired in childhood. Clinically, HZ manifests as a painful, vesicular rash with a dermatomal distribution. The risk of viral reactivation increases significantly with advancing age, immunosenescence, and, most notably, in the setting of acquired or iatrogenic immunosuppression [1].

The clinical burden of HZ extends well beyond its cutaneous presentation. Complications such as postherpetic neuralgia (PHN)—a chronic and often debilitating neuropathic pain syndrome—secondary bacterial superinfections, ophthalmic or neurological involvement, and, in rare cases, disseminated or visceral disease may result in substantial morbidity, reduced quality of life, and increased healthcare resource utilization [2, 3].

In the general population, the lifetime risk of developing HZ is approximately 30%, with incidence rates increasing sharply among older adults and individuals with chronic comorbidities such as diabetes and chronic obstructive pulmonary disease. However, individuals with hematological disorders—particularly those with malignancies or undergoing hematopoietic stem cell transplantation (HSCT)—are at substantially greater risk. In these patients, the combination of disease‐related immunosuppression, cytotoxic therapy, and delayed immune reconstitution following chemotherapy or transplantation creates a highly permissive environment for VZV reactivation and severe disease [4].

In addition to VZV, other herpesviruses—including herpes simplex virus (HSV), cytomegalovirus (CMV), Epstein–Barr virus (EBV), and human herpesvirus 6 (HHV‐6)—can cause significant morbidity in these immunocompromised patients. Reactivation of these viruses may further complicate the clinical management, particularly in the context of allogeneic HSCT, where they can lead to life‐threatening complications such as viral pneumonitis, encephalitis, or disseminated systemic infections. CMV, in particular, remains a major concern in HSCT recipients, and prophylaxis with antiviral agents like letermovir or (val‐)ganciclovir is routinely employed to reduce the risk of reactivation and associated morbidity. Importantly, the concurrent management of multiple herpesvirus reactivations must be carefully integrated with other preventive strategies, including vaccination, to optimize overall patient outcomes.

Over the past two decades, advances in antiviral prophylaxis and, more recently, the introduction of the adjuvanted recombinant zoster vaccine (aRZV; Shingrix) have significantly enhanced preventive strategies for immunocompromised individuals [5]. Nevertheless, important clinical uncertainties remain—particularly regarding the optimal timing, duration, and integration of antiviral prophylaxis with immunization.

This review synthesizes current evidence and guideline recommendations for the prevention of HZ in patients with hematological diseases, with a focus on high‐risk subgroups. It also explores practical implementation challenges, ongoing controversies, and key areas requiring further research to support evidence‐based, individualized prevention strategies.

To address these objectives, we performed a structured literature search in PubMed, Scopus, and Web of Science for articles published in English between 2002 and 2025. The following combinations of Medical Subject Headings (MeSH) and keywords were applied: “Herpes zoster,” “varicella‐zoster virus,” “hematological malignancies,” “stem cell transplant,” “antiviral prophylaxis,” “recombinant zoster vaccine,” “mRNA vaccines,” and “immunocompromised hosts.” Additional references were identified through manual screening of bibliographies from relevant original articles and review papers.

## 2 Pathogenesis of Herpes Zoster in Hematological Disorders

The pathogenesis of HZ is fundamentally driven by the loss or dysfunction of VZV‐specific cell‐mediated immunity. Following primary infection—typically occurring in childhood and manifesting as varicella (chickenpox)—VZV establishes lifelong latency within sensory neuronal ganglia. The maintenance of this latent state relies heavily on robust virus‐specific T‐cell responses that provide immunosurveillance and suppress viral reactivation.

In individuals with hematological disorders, this delicate immunological equilibrium is frequently disrupted by both the underlying disease and its treatment. The malignant process itself contributes to immune dysregulation through multiple mechanisms; for example, in chronic lymphoproliferative diseases, including chronic lymphocytic leukemia and indolent non‐Hodgkin lymphomas, the accumulation of dysfunctional lymphocytes further compromises immune competence [6].

Therapeutic interventions further exacerbate this immunosuppressive milieu. Cytotoxic chemotherapy causes profound and often prolonged lymphopenia, undermining cell‐mediated immunity. Monoclonal antibodies targeting surface antigens—such as CD20, CD19, and CD38—result in the depletion of essential immune cell subsets, including B cells, plasma cells, and natural killer cells, thereby impairing both humoral and cellular immunity. Targeted agents, such as JAK inhibitors (e.g., ruxolitinib) and proteasome inhibitors (e.g., bortezomib), disrupt immune signaling pathways and alter immune homeostasis, further increasing susceptibility to viral reactivation.

Among all therapeutic scenarios, HSCT is associated with the most profound and sustained immunosuppression. In autologous HSCT, although immune reconstitution occurs more rapidly than in allogeneic settings, it remains incomplete for several months post‐transplant. In allogeneic HSCT recipients, T‐cell and B‐cell recovery may be delayed for a year or more, particularly in the presence of graft‐versus‐host disease (GVHD) or prolonged immunosuppressive therapy. This population is therefore at exceptionally high risk for opportunistic infections, including reactivation of VZV and disseminated forms of HZ [4].

Overall, the reactivation of VZV in hematological patients reflects a complex interplay between host factors, disease‐related immune dysfunction, therapy‐induced immunosuppression, and impaired immune reconstitution. This complex pathophysiology underscores the need for targeted preventive strategies, including antiviral prophylaxis and vaccination, to reduce the substantial burden of HZ in this vulnerable population.

## 3 Epidemiology and Risk Factors

The baseline risk of HZ in the general population is primarily influenced by age and the progressive decline of immune function. The annual incidence is estimated at 2–4 cases per 1000 person‐years in adults under 50 years of age. This figure rises to 7–8 per 1000 in individuals over 50 years and exceeds 10 per 1000 among those aged 80 years or older, reflecting the impact of immune senescence on VZV reactivation [7, 8].

A comprehensive meta‐analysis, which screened 4417 studies and included 88 eligible publications comprising data on 3,768,691 HZ cases, confirmed immunosuppression as a dominant risk factor. Individuals living with HIV/AIDS exhibited the highest relative risk (RR) for HZ compared to immunocompetent controls (RR 3.22; 95% confidence interval [CI], 2.40–4.33), followed by patients with malignancies (RR 2.17; 95% CI, 1.86–2.53). A family history of HZ was also strongly associated with increased susceptibility (RR 2.48; 95% CI, 1.70–3.60), suggesting a potential genetic or shared environmental predisposition [9].

Additional risk factors included physical trauma (RR 2.01; 95% CI, 1.39–2.91) and advancing age (RR 1.65; 95% CI, 1.37–1.97). Chronic medical conditions—such as diabetes mellitus, rheumatoid arthritis, systemic lupus erythematosus, inflammatory bowel disease, cardiovascular disease, and chronic kidney disease—were also associated with modestly elevated risk, contributing to incidence rates between 4 and 11 per 1000 person‐years [10]. Female sex and psychological stress were identified as additional contributors, with relative risks ranging from 1.23 to 2.08. Interestingly, individuals identifying as Black had a lower risk of developing HZ (RR 0.69; 95% CI, 0.56–0.85), a finding that may reflect underlying immunogenetic or socioeconomic differences and warrants further investigation [9].

By contrast, the epidemiology of HZ changes significantly in immunosuppressed populations. Among recipients of solid organ transplants (SOT), incidence rates range from 12.1 to 78.8 per 1000 person‐years, depending on the transplanted organ, intensity of immunosuppression, and patient age. Kidney and lung transplant recipients over 60 years of age show the highest susceptibility [8, 11].

HSCT—both autologous and allogeneic—carries among the highest known risks for HZ reactivation. Incidence estimates ranging from 37.2 to 56.1 per 1000 person‐years, with the greatest risk occurring in the early post‐transplant period, coinciding with profound T‐cell depletion and delayed immune reconstitution. In allogeneic HSCT recipients, those with chronic GVHD or ongoing immunosuppressive therapy remain at elevated risk for years after transplantation.

Outside the transplant context, several hematological therapies are independently associated with increased HZ risk. These include proteasome inhibitors (e.g., bortezomib), JAK2 inhibitors (e.g., ruxolitinib), prolonged corticosteroid use, and lymphocyte‐depleting monoclonal antibodies targeting CD20, CD19, or CD52 [4, 12]. Moreover, persistent lymphopenia and hypogammaglobulinemia—common in chronic lymphoproliferative disorders such as chronic lymphocytic leukemia and multiple myeloma—further compound the susceptibility to reactivation risk.

Table 1 summarizes representative HZ incidence rates across various populations, illustrating a clear gradient of increasing risk in relation to age, comorbidities, and degree of immunosuppression. Notably, patients with hematologic malignancies or undergoing transplantation experience rates that far exceed those observed in the general population, reinforcing the need for robust preventive strategies.

**TABLE 1:** Representative incidence rates for herpes zoster across populations.

Population	Incidence per 1000 person‐years
General (age < 50)	2–4
Age > 80	> 10
Diabetes	4.3–9.4
COPD	7.4–11.4
Solid Organ Transplant	12.1–78.8
HSCT	37.2–56.1
Hematologic Neoplasia	2.9–32.0

The clinical consequences of HZ in hematological patients are particularly severe. Immunocompromised individuals are not only more likely to develop HZ, but they also face an elevated risk of complications. Lesions may extend beyond a single dermatome, and the likelihood of disseminated cutaneous or visceral involvement—including hepatitis, pneumonitis, and encephalitis—is significantly increased. Hospitalization rates are consequently higher, and active infection frequently results in treatment delays, compromising optimal hematological malignancies management.

Furthermore, the incidence of PHN—a debilitating neuropathic complication—is considerably greater in hematologic patients compared to immunocompetent individuals. In this population, PHN tends to be more severe, longer lasting, and less responsive to conventional therapies [1, 2, 3]. Additional complications, such as secondary bacterial infections, non‐healing ulcers, and chronic pain syndromes further increase morbidity and place a substantial burden on both the patient and the healthcare system.

## 4 Evidence for Antiviral Prophylaxis

Antiviral prophylaxis (AVP) with acyclovir or valacyclovir remains a cornerstone in the prevention of HZ among patients with hematological disorders. Randomized trials and meta‐analyses have consistently demonstrated that AVP significantly reduces the incidence of HZ during periods of profound immunosuppression, particularly in the context of HSCT [13].

Table 2 summarizes the current AVP strategies recommended across a range of hematologic clinical scenarios, including both transplant and non‐transplant settings. The decision to initiate prophylaxis is primarily driven by the intensity and anticipated duration of immunosuppression, which varies based on disease subtype, treatment modality, and the timeline of immune reconstitution.

**TABLE 2:** Antiviral prophylaxis in hematological disorders and transplant settings.

Clinical scenario	Antiviral prophylaxis (AVP)	Key notes
Autologous HSCT	Acyclovir 800 mg BID or Valacyclovir 500–1000 mg BID from start of conditioning through ≥ 12 months post‐transplant	Extend AVP if ongoing immunosuppression or delayed immune recovery
Allogeneic HSCT	Same agents and doses as above; duration often > 12 months, extended in the presence of GVHD or ongoing immunosuppression	Prolonged AVP may be required in patients with chronic GVHD or incomplete immune reconstitution
Proteasome Inhibitor Therapy (e.g., MM)	Acyclovir 400–800 mg BID or Valacyclovir 500 mg BID during treatment and for ≥ 1 month after cessation	Continue AVP during maintenance therapy, including bispecific antibodies
JAK Inhibitor Therapy (e.g., MPN)	Acyclovir or Valacyclovir throughout treatment and ≥ 1 month after discontinuation	Assess the need for prolonged prophylaxis based on immune status and patient age
CAR‐T Cell Therapy	Acyclovir 800 mg BID or Valacyclovir 500 mg BID from lymphodepletion until at least 6 months post‐infusion	Duration may be extended in case of prolonged B‐cell aplasia or ongoing hypogammaglobulinemia
Bispecific Antibody or Novel Therapy	No standard; consider AVP during lymphopenia or if prior heavily immunosuppressive therapy	Individualize based on immune reconstitution and comorbidities; limited prospective data available
Lymphoproliferative Disorders (e.g., CLL, lymphoma)	AVP during and after fludarabine, bendamustine, or prolonged corticosteroids	Consider at diagnosis or before therapy in the elderly; extend AVP in patients with persistent lymphopenia
Hematologic Malignancies Not Undergoing HSCT	Acyclovir 400–800 mg BID or Valacyclovir 500 mg BID during therapy and for ≥ 6 months thereafter	Consider prophylaxis with lymphotoxic chemotherapy (e.g., anti‐CD20, purine analogs, alkylators)
Non‐malignant Hematologic Disease in the Elderly	Consider AVP during steroid therapy lasting ≥ 2 months	Initiate early in frail or elderly patients with comorbidities
Breakthrough or Resistant VZV Infection	Escalate to IV Acyclovir 10 mg/kg q8h or Foscarnet IV per institutional protocol	Requires virologic confirmation; manage as disseminated infection in an immunocompromised host

Abbreviations: AVP, antiviral prophylaxis; BID, twice daily; CAR‐T, chimeric antigen receptor T‐cell therapy; CLL, chronic lymphocytic leukemia; GVHD, graft‐versus‐host disease; HSCT, hematopoietic stem cell transplantation; MM, multiple myeloma; MPN, myeloproliferative neoplasms.

In autologous HSCT, prophylactic administration of acyclovir (800 mg twice daily) or valacyclovir (500–1000 mg twice daily) is typically initiated with conditioning therapy and continued for at least 12 months following transplant. This approach is supported by high‐quality evidence demonstrating substantial reductions in HZ incidence, particularly during the first post‐transplant year when T‐cell immunitity remains partially reconstituted [14]. In allogeneic HSCT recipients, similar regimens are employed; however, the duration of AVP is often extended beyond 12 months in the presence of GVHD or ongoing systemic immunosuppression. These patients experience prolonged deficits in T‐cell immunity, placing them at sustained risk for VZV reactivation [4]. These individuals are at sustained risk for VZV reactivation due to impaired immune reconstitution and dysfunctional T‐cell recovery [4].

Outside of the transplant context, various therapeutic classes necessitate AVP due to their immunosuppressive profiles. In multiple myeloma, use of proteasome inhibitors—particularly bortezomib—has been strongly associated with a significantly increased risk of VZV reactivation. Randomized clinical trials and real‐world data support routine antiviral prophylaxis throughout active treatment and for at least 1 month following its discontinuation [15]. AVP should also be continued during maintenance therapy, especially in patients receiving bispecific antibodies or anti‐BCMA agents, which may induce prolonged immunosuppression and delay immune reconstitution.

Patients with myeloproliferative neoplasms treated with JAK2 inhibitors, such as ruxolitinib, also benefit from AVP. These agents impair both innate and adaptive immunity by suppressing dendritic cell function and natural killer cell activity. Prophylaxis with acyclovir or valacyclovir is recommended throughout the course of therapy and for at least 1 month thereafter, with extended use advised in older or medically complex patients [12].

Chimeric antigen receptor T‐cell (CAR‐T) therapy has introduced new complexities in infection prevention. Recipients frequently develop prolonged B‐cell aplasia and impaired T‐cell function, compounded by lymphodepleting chemotherapy and adjunctive immunomodulatory agents such as corticosteroids and tocilizumab. Current best practice recommends initiating AVP during the lymphodepletion phase and continuing for a minimum of 6 months post‐infusion. Prophylaxis may be extended in the presence of persistent hypogammaglobulinemia, lymphopenia, or ongoing immunosuppression [4, 16].

Patients receiving bispecific antibodies, immune checkpoint inhibitors, or other novel immunotherapies should be assessed individually for AVP. Although formal guidelines are evolving, decisions should consider immune markers (e.g., CD4+ lymphocyte count, serum immunoglobulin levels) prior iVZV history, and concurrent therapies [4].

Chronic lymphocytic leukemia or indolent lymphomas also confer substantial risk for HZ due to both disease‐associated immune dysfunction and the lymphodepleting effects of agents such as fludarabine, bendamustine, and anti‐CD20 monoclonal antibodies (e.g., rituximab). AVP is strongly advised throughout therapy and for several months after its completion, particularly in patients with persistent lymphopenia or hypogammaglobulinemia.

An often underrecognized at‐risk population includes elderly patients with non‐malignant hematologic disorders, such as autoimmune cytopenias, who are treated with prolonged corticosteroids or immunosuppressive agents. Patients aged ≥ 70 years, or those with multiple comorbidities, face a higher risk for severe VZV reactivation and may benefit from AVP even in the absence of malignancy.

Despite the efficacy of prophylaxis, breakthrough HZ infections may occur in severely immunocompromised patients. In such cases, prompt virological confirmation is essential, and treatment should be escalated to intravenous antiviral therapy such as acyclovir (10 mg/kg every 8 h) or foscarnet. These infections should be treated as disseminated HZ, requiring hospitalization and close clinical monitoring [17].

Renal dose adjustments is essential, as both acyclovir and valacyclovir are eliminated via the kidneys. While generally well‐tolerated, rare adverse effects such as nephrotoxicity and neurotoxicity can occur, particularly in elderly or renally impaired patients. Discontinuation of AVP should be guided by immune recovery markers, including CD4+ counts (preferably > 200/μL), and must be tailored to the patient's treatment history risk profile.

In summary, antiviral prophylaxis remains a key strategy in the prevention of HZ in hematological patients. Its implementation must be risk‐adapted and personalized, accounting for the degree and duration of immunosuppression, speed of immune reconstitution, and the evolving therapeutic landscape. As modern hematology increasingly incorporates CAR‐T, bispecific antibodies, and immune checkpoint inhibitors into clinical practice, AVP strategies must evolve in parallel to ensure.

## 5 Immunization Strategies Against Herpes Zoster in Hematological Patients

Immunization against HZ has undergone substantial evolution over the past two decades, driven by increasing awareness of the morbidity associated with VZV reactivation—particularly among older adults and immunocompromised population. Patients with hematological disorders represent a key target group for vaccination, given their heightened risk of severe and prolonged disease, including complications such as PHN and disseminated infection and treatment delays.

Historically, the only available vaccine for HZ prevention was the live‐attenuated zoster vaccine (ZVL, Zostavax), derived from the Oka strain and licensed in 2006. ZVL demonstrated moderate efficacy in immunocompetent adults, with a 51% reduction in incidence and a 67% reduction in PHN, as shown in a pivotal randomized controlled trial [18]. However, due to the theoretical risk of vaccine‐strain reactivation, ZVL was contraindicated in immunocompromised individuals, including patients with hematological malignancies or who had undergone stem cell transplantation.

ProVarivax, a live‐attenuated vaccine derived from the Oka strain, has been widely used for the prevention of primary varicella infection in children and adults as part of routine vaccination programs. Although not specifically designed for the prevention of herpes zoster prevention, its use indirectly reduces the incidence of zoster in children and young adults by preventing initial varicella infection and thereby lowering the risk of subsequent reactivation. Similar to Zostavax, ProVarivax is considered safe for immunocompetent individuals; however, its administration in immunocompromised patients is not recommended because of the potential risk of vaccine strain reactivation.

The approval of the recombinant zoster vaccine (RZV, Shingrix) in 2017 marked a major advance in HZ prevention, particularly for high‐risk and immunosuppressed populations. RZV is a non‐live, adjuvanted subunit vaccine composed of the VZV glycoprotein E (gE) and the AS01B adjuvant system, designed to induce strong antigen‐specific T‐cell and humoral responses even in settings of impaired immunity. In the large phase III trials ZOE‐50 and ZOE‐70, RZV demonstrated 97.2% efficacy in adults aged ≥ 50 years and 89.8% efficacy in those aged ≥ 70 years, with durable protection observed for at least a decade [19, 20].

Subsequent trials and real‐world studies have confirmed that RZV is both immunogenic and safe in immunocompromised patients, including those with hematologic malignancies. In a randomized controlled study involving HSCT recipients, RZV induced robust gE‐specific CD4+ T‐cell responses and conferred 68.2% protection against HZ [21]. Similar immunogenicity and safety profiles have been observed in patients with solid tumors [22, 23] and in people living with HIV, in whom the vaccine enhanced VZV‐specific immunity without significant safety concerns [24].

Current guidance from the U.S. Centers for Disease Control and Prevention (CDC) and the Advisory Committee on Immunization Practices (ACIP) recommend RZV for all adults aged ≥ 18 years who are, or will become, immunocompromised due to disease or therapy, including patients with hematologic cancers [25, 26].

The optimal timing of RZV administration remains an area of active investigation. Ideally, vaccination should occur during phases of immune recovery, such post‐chemotherapy or between treatment cycles, to maximize vaccine responsiveness. Nevertheless, emerging evidence suggests that RZV is immunogenic even when administered during active chemotherapy or maintenance therapy, supporting its use across broader clinical spectrum [27].

A comprehensive systematic review by Williams et al. summarized RZV immunogenicity and effectiveness in both immunocompetent and immunocompromised populations [28]. In immunocompetent adults aged ≥ 50 years, RZV efficacy ranged from 90% to 97% in clinical trials, with real‐world effectiveness estimates of 71%–86%. Crucially, protection remained durable for at least 10 years, with little variation by age, sex, or ethnicity. In several studies efficacy against complications—exceeded 90%.

Durability of protection emerged as a key strength of RZV. Both antibody titers and cell‐mediated immunity persisted for 10 years or longer, with modeling suggesting that protection may last up to 20 years. RZV's safety profile has also been favorable: while mild to moderate local and systemic reactogenicity (e.g., injection site pain, fatigue) are common, serious adverse events remain rare and comparable to placebo. Post‐marketing surveillance has not identified a causal relationship between RZV and severe events such as Guillain–Barré syndrome.

From a public health perspective, the RZV's compatibility with other adult vaccines, including influenza, pneumococcal, and COVID‐19 vaccines, facilitates its integration into routine immunization schedules, particularly in patients with hematological malignancies who often require frequent clinic visits.

Nonetheless, several critical challenges remain. Data from low‐ and middle‐income countries are limited, despite potential epidemiologic and healthcare infrastructure differences. The high cost of RZV may limit widespread uptake, emphasizing the need for cost‐effectiveness evaluations in diverse settings. Additionally, further research is warranted to define optimal dosing schedules and long‐term protection in patients receiving repeated immunosuppressive therapies or undergoing CAR‐T or bispecific antibody treatments.

Table 3 summarizes outcomes from key RZV studies across both immunocompetent and immunocompromised populations. Collectively, these findings support the widespread adoption of RZV as a safe and highly effective preventive measure against herpes zoster in patients with hematological disorders.

**TABLE 3:** Summary of recombinant zoster vaccine (RZV) main outcomes in immunocompetent versus immunocompromised patients.

Parameter	Immunocompetent patients	Immunocompromised patients
Vaccine Efficacy (RCTs)	90%–97% (ZOE‐50/70 trials, mean follow‐up 3.5–3.7 years)	68%–87% (e.g., 68% in HSCT; up to 87% in hematological malignancies)
Vaccine Effectiveness	71%–86% (Real‐world studies, 0.7–3.1 year follow‐up)	61%–100% (Effectiveness lower than in immunocompetent, but substantial)
Protection Duration	> 70% protection sustained ≥ 10 years (Long‐term follow‐up studies)	Data limited, but moderate protection observed for up to several years
Efficacy Against PHN	> 90% (91.2% pooled analysis, > 50 years old)	Data is limited, but a significant reduction in complications is observed
Efficacy Against HZO	> 90% (93.7% against non‐PHN complications; real‐world: 67%–93%)	Not specifically reported; presumed benefit
Hospitalization	No cases in vaccine arm (ZOE‐50/70); high protection (100%, wide CI)	Lower hazard ratio of hospitalization (HR 0.15, 95% CI 0.03–0.68 in HSCT)
Immunogenicity	Robust humoral and cell‐mediated responses sustained ≥ 10 years	Robust, but generally lower responses; still marked improvement over baseline
Co‐administration	Safe and immunogenic with influenza, pneumococcal, and COVID‐19 vaccines	Not specifically reported; presumed similar
Safety	Increased local/systemic reactions versus placebo, mostly mild/short‐lived Serious events are rare and similar to a placebo	Similar profile; no increase in severe adverse events versus placebo
Age/Ethnicity/Sex Impact	No significant differences across subgroups	Not enough data for subgroup analysis
Cost‐Effectiveness	High in most high‐income settings; key consideration for policy	Not well studied; more data needed
Key Remarks	Long‐lasting, high protection; suitable for routine use in older adults	Effective option where live vaccine is contraindicated; needs more data

Abbreviations: CI, Confidence interval; HSCT, Hematopoietic stem cell transplant; HZO, Herpes zoster ophthalmicus; PHN, Post‐herpetic neuralgia; RCT, Randomized controlled trial.

## 6 Advances in Vaccination

The advent of the adjuvanted recombinant zoster vaccine (aRZV, Shingrix) has markedly improved the prevention in immunocompromised populations. Unlike the live‐attenuated zoster vaccine (ZVL, Zostavax), which is contraindicated in most immunosuppressed patients due to the risk of vaccine‐strain reactivation, aRZV is both safe and highly immunogenic in these high‐risk groups [21].

Phase III trials involving patients with hematological malignancies and HSCT recipients have demonstrated that aRZV reduces HZ incidence by 68% to 87% compared with placebo. These studies also revealed that the vaccine elicits robust humoral and cellular immune responses, even in individuals recently transplanted or receiving active immunosuppressive therapy [29].

Recently, mRNA‐based vaccine platforms—pivotal in the rapid development of the COVID‐19 vaccines—have emerged as promising tools for future HZ prevention. These platforms offer several theoretical and practical advantages: rapid development, scalable production, strong induction of cellular immunity without the need for complex adjuvants, and adaptability to antigenic evolution. Preclinical data are particularly encouraging [30]. A glycoprotein E (gE)‐based mRNA vaccine has demonstrated humoral immunogenicity on par with Shingrix, while exceeding both currently licensed HZ vaccines in the magnitude of cellular immune responses.

This is a critical advantage, as durable cell‐mediated immunity is believed to be essential for controlling VZV reactivation, particularly in older adults and immunocompromised hosts. Furthermore, the mRNA vaccine demonstrated potent Fc‐mediated effector functions, enhancing its potential for clinical efficacy. Although these findings stem from murine models—which do not fully replicate human immune responses or VZV pathogenesis—they provide a compelling rationale for continued development. Importantly, mRNA‐based vaccines could alleviate some of the logistical and reactogenicity challenges associated with aRZV, particularly if formulated without rare or highly reactogenic adjuvant components. Their production flexibility may also help address global supply limitations, expanding accessibility in both high‐ and low‐resource settings.

Future research should aim to validate these preliminary findings in non‐human primate models and early‐phase human clinical trials. Key priorities include assessing the durability of immune protection, vaccine safety, tolerability, and reactogenicity—especially in elderly individuals and those undergoing immunosuppressive therapies. If the strong immunogenicity observed in preclinical models is confirmed in clinical settings, an mRNA‐based HZ vaccine could represent a paradigm shift in varicella‐zoster prevention, combining enhanced efficacy, improved tolerability, and scalable manufacturing capabilities.

## 7 Conclusion

HZ remains a serious and largely preventable complication in patients with hematological diseases, primarily due to deficits in cell‐mediated immunity arising from the underlying malignancy, therapeutic interventions, or transplant‐associated immune suppression. Beyond its characteristic vesicular eruption, HZ is associated with considerable morbidity, including prolonged neuropathic pain, ophthalmic complications, and functional decline. Given the immunological vulnerability of this population, preventive strategies combining AVP and immunization are essential components of comprehensive care.

The aRZV has emerged as the cornerstone of HZ vaccination in immunocompromised individuals. As a non‐live vaccine, aRZV provides a safer alternative to the previously available live‐attenuated vaccine (Zostavax), which is contraindicated in most immunocompromised hosts. Phase III trials in patients with hematological malignancies and recipients of HSCT have shown that aRZV significantly reduces HZ incidence and elicits robust humoral and cellular immune responses, even when administered during or shortly after chemotherapy or transplant [29].

Reflecting this strong evidence base, updated recommendations from the U.S. Advisory Committee on Immunization Practices (ACIP) now endorse aRZV for immunocompromised adults aged ≥ 18 years, including those with hematological malignancies, HSCT recipients, and patients receiving B‐cell–depleting therapies. The vaccine is administered in a two‐dose series, with the first dose typically initiated 2 to 6 months post‐transplant or after completion of intensive immunosuppressive therapy, depending on the patient's immune reconstitution status. Importantly, ACIP guidelines affirm that coadministration with antiviral prophylaxis is acceptable and does not compromise vaccine efficacy, addressing a key concern in hematology practice [25].

Recent clinical guidelines from infectious disease and hematology societies further support the integration of both AVP and aRZV into HZ prevention protocols across a broad spectrum of hematologic settings. As summarized in Table 4, patients undergoing autologous or allogeneic HSCT should receive AVP for ≥ 12 months post‐transplant, with aRZV initiated at 2 to 6 months thereafter, depending on GVHD status and T‐cell reconstitution. Similar approaches are endorsed for patients receiving proteasome inhibitors, JAK inhibitors, or immunochemotherapy with fludarabine or bendamustine. Even in elderly patients with non‐malignant hematologic disorders receiving chronic corticosteroids, individualized prophylactic strategies—including both AVP and aRZV—should be considered based on immunological risk profiles [4].

**TABLE 4:** Summary of guideline recommendations for HZ prevention in hematological disorders.

Clinical scenario	AVP recommendation	aRZV recommendation
Autologous HSCT	Yes, ≥ 1 year	2 months post‐transplant
Allogeneic HSCT	Yes, ≥ 1 year; longer if GVHD	≥ 6 months post‐transplant, individualized
Proteasome inhibitor therapy (MM)	Yes, during therapy + 1 month after	Before therapy, if possible, or after
JAK inhibitor therapy (MPN)	Yes, during therapy + 1 month after	Before therapy, if possible, or after
Lymphoproliferative disorders	Yes, during/after fludarabine/bendamustine	At diagnosis or before therapy (elderly)
Non‐malignant hematologic disease	Consider in elderly on steroids	At diagnosis (elderly)

The emergence of mRNA‐based zoster vaccines introduces an exciting new frontier. These platforms offer the potential for enhanced immunogenicity, simplified production, and reduced reliance on reactogenic adjuvant components. Preliminary murine studies have demonstrated superior T‐cell responses compared to existing vaccine platforms, with encouraging implications for durable protection in immunocompromised patients [30]. Human trials will be essential to confirm these findings and to assess safety and tolerability, particularly in vulnerable populations with hematologic conditions or undergoing immunosuppressive therapies.

In conclusion, HZ prevention in hematological patients has entered a new era, guided by evidence‐based protocols that combine AVP and recombinant vaccination. As supported by ACIP and international hematology guidelines, timely and individualized prophylaxis—tailored to treatment intensity and immune status—can substantially reduce the burden of HZ in this population. Ongoing research into long‐term vaccine effectiveness, mRNA‐based technologies, and real‐world implementation will be critical for optimizing outcomes. Ultimately, integrating antiviral and vaccine‐based preventive strategies into routine hematologic care is not only feasible but imperative to reduce HZ–related morbidity and improve patient quality of life.

## Author Contributions

**Enrica Antonia Martino, Fortunato Morabito, and Massimo Gentile:** conceptualization. **Ernesto Vigna, Antonella Bruzzese, and Fortunato Morabito:** methodology. **Santino Caserta:** writing – original draft preparation. **Massimo Gentile and Fortunato Morabito:** writing – review and editing. All authors have read and agreed to the published version of the manuscript.

## Conflicts of Interest

The authors declare no conflicts of interest.

## Data Availability

Data sharing not applicable to this article as no datasets were generated or analyzed during the current study.
